# 2-Fluoro-L-Fucose Is a Metabolically Incorporated Inhibitor of Plant Cell Wall Polysaccharide Fucosylation

**DOI:** 10.1371/journal.pone.0139091

**Published:** 2015-09-28

**Authors:** Jose A. Villalobos, Bo R. Yi, Ian S. Wallace

**Affiliations:** Department of Biochemistry and Molecular Biology, University of Nevada, Reno, Reno, Nevada, 89557, United States of America; The University of Melbourne, AUSTRALIA

## Abstract

The monosaccharide L-fucose (L-Fuc) is a common component of plant cell wall polysaccharides and other plant glycans, including the hemicellulose xyloglucan, pectic rhamnogalacturonan-I (RG-I) and rhamnogalacturonan-II (RG-II), arabinogalactan proteins, and N-linked glycans. Mutations compromising the biosynthesis of many plant cell wall polysaccharides are lethal, and as a result, small molecule inhibitors of plant cell wall polysaccharide biosynthesis have been developed because these molecules can be applied at defined concentrations and developmental stages. In this study, we characterize novel small molecule inhibitors of plant fucosylation. 2-fluoro-L-fucose (2F-Fuc) analogs caused severe growth phenotypes when applied to Arabidopsis seedlings, including reduced root growth and altered root morphology. These phenotypic defects were dependent upon the L-Fuc salvage pathway enzyme L-Fucose Kinase/ GDP-L-Fucose Pyrophosphorylase (FKGP), suggesting that 2F-Fuc is metabolically converted to the sugar nucleotide GDP-2F-Fuc, which serves as the active inhibitory molecule. The L-Fuc content of cell wall matrix polysaccharides was reduced in plants treated with 2F-Fuc, suggesting that this molecule inhibits the incorporation of L-Fuc into these polysaccharides. Additionally, phenotypic defects induced by 2F-Fuc treatment could be partially relieved by the exogenous application of boric acid, suggesting that 2F-Fuc inhibits RG-II biosynthesis. Overall, the results presented here suggest that 2F-Fuc is a metabolically incorporated inhibitor of plant cellular fucosylation events, and potentially suggest that other 2-fluorinated monosaccharides could serve as useful chemical probes for the inhibition of cell wall polysaccharide biosynthesis.

## Introduction

Cell walls are polysaccharide-rich extracellular matrices that surround all plant cells and critically influence growth and development. Collectively, cell wall polysaccharides represent the most abundant biopolymers in nature and are the largest renewable source of food, fiber, fuel, and textiles for human and forage animal utilization [1]. Cell wall polysaccharides are structurally heterogenous [2–4], but can generally be grouped into three functional categories: cellulose, neutral hemicelluloses, and acidic pectins. The proper biosynthesis, deposition, and organization of these cell wall polysaccharides fundamentally influences basic cellular processes, such as cell division, cell expansion, and the acquisition of cell shape [2, 3].

In *Arabidopsis thaliana*, the monosaccharide L-fucose (L-Fuc) is a common component of plant cell wall polysaccharides and other cellular glycans [5, 6], including xyloglucan [7–9], soybean pectic rhamnogalacuronan-I (RG-I) [10], rhamnogalacturonan-II (RG-II) [11–13], arabinogalactan proteins [14, 15], and N-linked glycans [16]. Using GDP-L-Fuc as a substrate, FUT family glycosyltransferases specifically transfer L-Fuc to these polysaccharides [14, 16–18]. GDP-L-Fuc is synthesized by two distinct pathways in Arabidopsis. In the *de novo* biosynthesis pathway, GDP-D-mannose is converted to GDP-L-Fuc through the sequential action of the GDP-D-mannose-4,6-dehydratase MUR1 [19], and the bifunctional 3,5-epimerase-4-reductase GER1 [20]. Additionally, GDP-L-Fuc can be synthesized from free L-Fuc through the action of the bifunctional enzyme L-Fucose Kinase/ GDP-L-Fucose Pyrophosphorylase (FKGP) [21]. Genetic analysis of Arabidopsis *mur1* mutants revealed a critical role for L-Fuc biosynthesis in plant growth and development. These mutants are dwarfed, exhibit reduced cell expansion in aerial tissues, and reduced cell wall fucose content [19, 22]. Further investigation revealed that the structure of RG-II was altered in the *mur1* mutant. RG-II is a complex pectic polysaccharide with a highly conserved structure consisting of five side chains named A-E [13, 23]. Side chains A and B typically contain L-Fuc residues, and these monosaccharides are replaced with L-galactose in the *mur1* mutant [24]. Additionally, RG-II monomers can dimerize through the formation of borate esters between apiose residues in two RG-II monomers [25]. The *mur1* mutant also exhibits reduced boron-mediated RG-II dimerization, and this phenotype can be rescued by the addition of exogeneously applied boric acid [24, 26, 27].

Due to the essential role of cell wall biosynthesis in plant development, many null mutations in cell wall biosynthetic enzymes are lethal [28–33]. Well-characterized small molecule inhibitors of cell wall polysaccharide biosynthesis serve as important tools for cell wall characterization because these inhibitors can be applied at defined developmental stages and concentrations, thus avoiding lethality due to genetic disruption. Numerous phenotypic screens for small molecule inhibitors of cell wall biosynthesis have been conducted, and have identified a variety of cellulose biosynthesis inhibitors [34–37]. Small molecule inhibitors of xyloglucan endo-transglycosylase have also been identified [38]. However, these molecules are structurally diverse, precluding the facile identification of a direct target. Recently, a 2β-deoxy analog of 3-deoxy-2-D-manno-octulosonic acid (Kdo) was reported to inhibit CMP-Kdo synthase in Arabidopsis and subsequently impair RG-II biosynthesis [39], providing one of the first semi-rationally designed inhibitors affecting cell wall biosynthesis.

These observations open the possibility that rationally-designed monosaccharide analogs could be metabolically incorporated into plant cells to inhibit cell wall polysaccharide biosynthesis. To test this hypothesis, we screened numerous deoxy and fluro monosaccharide analogs of commonly occurring sugars in cell wall polysaccharides. We reasoned that deoxy monosaccharide analogs would serve as chain terminators by competing with the natural monosaccharide and inhibiting polysaccharide chain elongation due to the lack of one hydroxyl group required for glycosidic bond formation. Furthermore, we reasoned that fluorinated monosaccharide analogs would alter the interaction of sugar nucleotides with the active site of cognate glycosyltransferases and potentially inhibit cell wall polysaccharide biosynthesis. Here, we describe 2-flouro-L-fucose (2F-Fuc) as a potent inhibitor of cell wall polysaccharide fucosylation, and provide evidence that this monosaccharide analog is converted to GDP-2F-Fuc by the metabolic enzyme L-Fucose Kinase/ GDP-L-Fucose Pyrophosphorylase (FKGP). Chemical complementation experiments further suggest that while 2F-Fuc likely targets a variety of fucosylation events, the majority of phenotypic defects produced by this molecule are related to impaired biosynthesis of the pectic polysaccharide RG-II.

## Materials and Methods

### Monosaccharide analog growth assays

*Arabidopsis thaliana* Col-0 seeds were sterilized in 30% (v/v) sodium hypochlorite, 0.1% (w/v) sodium dodecyl sulfate for 20 minutes at 25°C. Seeds were washed five times in sterile water and incubated at 4°C for 48 hours before plating. Seeds were germinated on MS media (1/2X Murashige and Skoog salts, 10 mM MES-KOH pH 5.7, 1% [w/v] sucrose, and 1% [w/v] phytoagar) and grown vertically for 7 days under long day conditions (16 hr light/ 8 hr dark) at 22°C. Arabidopsis mutants harboring T-DNA insertions in xyloglucan fucosyltransferase (FUT1), N-linked glycan fucosyltransferases (FUT11, 12, and 13), and arabinogalactan protein fucosyltransferases (FUT4 and FUT6) were previously described elsewhere [15, 16, 18, 40]. Monosaccharide analogs were purchased from Carbosynth (Berkshire, UK). Analogs were diluted in DMSO and included in growth media at the indicated concentrations. Plants treated with 0.1% (v/v) DMSO served as negative controls. After 7 days of growth, seedlings were straightened on the treatment plate and scanned with a flatbed scanner. Root lengths were quantified using ImageJ (imagej.nih.gov/ij). For short-term treatment experiments, Col-0 seeds were sterilized, stratified, and plated on MS medium as described above. These seedlings were grown under long day conditions at 22°C for 4 days. Seedlings were then transferred to MS media with or without 100 μM 2F-Fuc and were incubated for an additional 5 days under the previously described growth conditions. For the first three days, root lengths were quantified every 12 hours using ImageJ. Five days after transfer, the root tips of representative seedlings were imaged with a Leica EZ4HD video dissecting scope at 35X magnification.

### FKGP knockout isolation

The Arabidopsis FKGP gene was queried against the Salk Institute T-DNA insertional mutant database [41], and several potential T-DNA lines were identified. These seed populations were propagated on MS media as described above and transplanted to soil after 14 days. Transplanted seedlings were maintained in a growth chamber at 22°C under long day conditions (16 hr light/ 8 hr dark) until maturity.

Genomic DNA isolation for PCR genotyping was performed essentially as previously described [42]. One leaf from each plant was removed and homogenized in a 1.5 mL Eppendorf tube with a Teflon pestle. Four hundred microliters of Edwards Buffer (200 mM Tris-HCl, 250 mM NaCl, 25 mM EDTA, 0.5% [w/v] SDS) was added to the homogenate, and the samples were incubated at 25°C for 1 hr. Samples were centrifuged at 12,000 x g for 10 min, and 300 μL of the supernatant was transferred to a new Eppendorf tube containing 300 μL of isopropanol. These samples were vortexed and centrifuged at 12,000 x g for 5 min. The supernatant was removed and 750 μL of 70% (v/v) ethanol was added. Samples were centrifuged for 3 min at 12,000 x g, the supernatant was removed, and the pellet was air dried for 20 min. The dried pellet was resuspended in 100 μL of sterile water.

Each genomic DNA sample was analyzed by genotyping PCR using locus specific primers (SALK_037697 LP: GTGCAAGACAAGCTTTCCAAG and SALK_037697 RP: CTAGTGGGACCTCCCAAAGAC) as well as a T-DNA specific left border primer (LBb1.3: ATTTTGCCGATTTCGGAAC), and ExTaq polymerase (Takara Bio, Mountain View, CA). Reactions were cycled under the following conditions: 95°C initial denaturation for 5 min, 35 cycles of 95°C for 30 sec, 52°C for 30 sec, 72°C for 1.5 min, final extension at 72°C for 7 min. The resulting PCR products were separated on 1.0% (w/v) agarose gels and documented with a Bio-Rad Gel Doc XR+ Image analysis workstation.

### Alditol acetate monosaccharide analysis

Plant material was placed in a preweighed screw cap microcentrifuge tube and incubated with 1.5 mL of 70% (v/v) ethanol for 6 hours at 25°C on a nutating shaker. The samples were centrifuged at 12,000 rpm for 10 minutes, and the supernatant was removed. An additional 1.5 mL of 70% ethanol was added and this wash step was repeated. The seedlings were then washed with 1.5 mL of 1:1 (v/v) chloroform: methanol for 6 hours at 25°C. The chloroform methanol supernatant was removed and samples were dried under a stream of air. Three steel balls were added to the dried plant material, and homogenized in a Retsch MM301 ball mill for 2.5 minutes at 25Hz. The steel balls were removed and the resulting alcohol insoluble residue (AIR) was weighed.

Five to ten mg of dry plant material was subjected to weak acid hydrolysis by adding 250 μL of 2M triflouro acetic acid (TFA) and 100 μg of D-myo-Inositol as an internal standard (MP Biomedicals). Samples were incubated at 121°C for 1.5 hours, and then centrifuged at 12,000 rpm for 10 minutes to pellet insoluble material. The supernatants were transferred to screw cap glass vials, and TFA was evaporated under a stream of N_2_ at 40°C. The resulting residue was dissolved in 250 μL of 2-propanol, vortexed and evaporated under a stream of N_2_ gas at 40°C. This process was repeated two additional times. Two hundred microliters of freshly prepared reducing solution (10 mg/mL NaBH_4;_ 1M NH_4_OH) was added to each sample and incubated at room 25°C for 1.5 hours. The reduction reaction was quenched with 150 μL of glacial acetic acid and an additional 250 μL of 9:1 (v/v) methanol:acetic acid was added. The liquid was dried under a stream of N_2_ gas at 40°C. The residue was washed with 250 μL of 9:1 methanol:acetic acid 2 additional times and dried under N_2_ gas at 40°C. The samples were washed by adding 250 μL of methanol and evaporated under N_2_ gas at 40°C. This process was repeated 4 times. Alditol acetylation was performed by adding 50 μL of pyridine and 50 μL of acetic anhydride to each sample. Samples were incubated in sealed glass tubes at 120°C for 20 minutes, then cooled on ice for 30 minutes. Solvent was evaporated under N_2_ gas at 25°C, and 200 μL of toluene was added to each sample and evaporated twice under N_2_ gas at 25°C. To this residue containing alditol acetates 0.5 mL of ethyl acetate and 2.0 mL of water was added. Samples were vortexed thoroughly and centrifuged at 500 rpm for 20 minutes. The upper organic phase was transferred into screw cap microcentrifuge tubes and evaporated under N_2_ gas at 25°C. Samples were prepared for gas chromatography by resuspending in 300 μL of acetone. One hundred microliters of each sample was transferred to GC vials and diluted with an additional 200 μL of acetone.

Samples were analyzed on an Agilent 7890A gas chromatography instrument (Agilent Technologies) equipped with a 30 mm x 0.25 mm x 0.25 μm SP-2380 fused silica capillary column (Supelco), under the following conditions: inlet temperature of 250°C, initial oven temperature 160°C held for 2 minutes, first temperature ramp at 20°C/min to 245°C, hold at 245°C for 12 minutes, second temperature ramp at 20°C/min to 270°C. Data was analyzed using GC Open Lab software (Agilent Technologies).

## Results

### Effect of fluoro and deoxy L-Fuc analogs on plant growth

**Fig 1:**
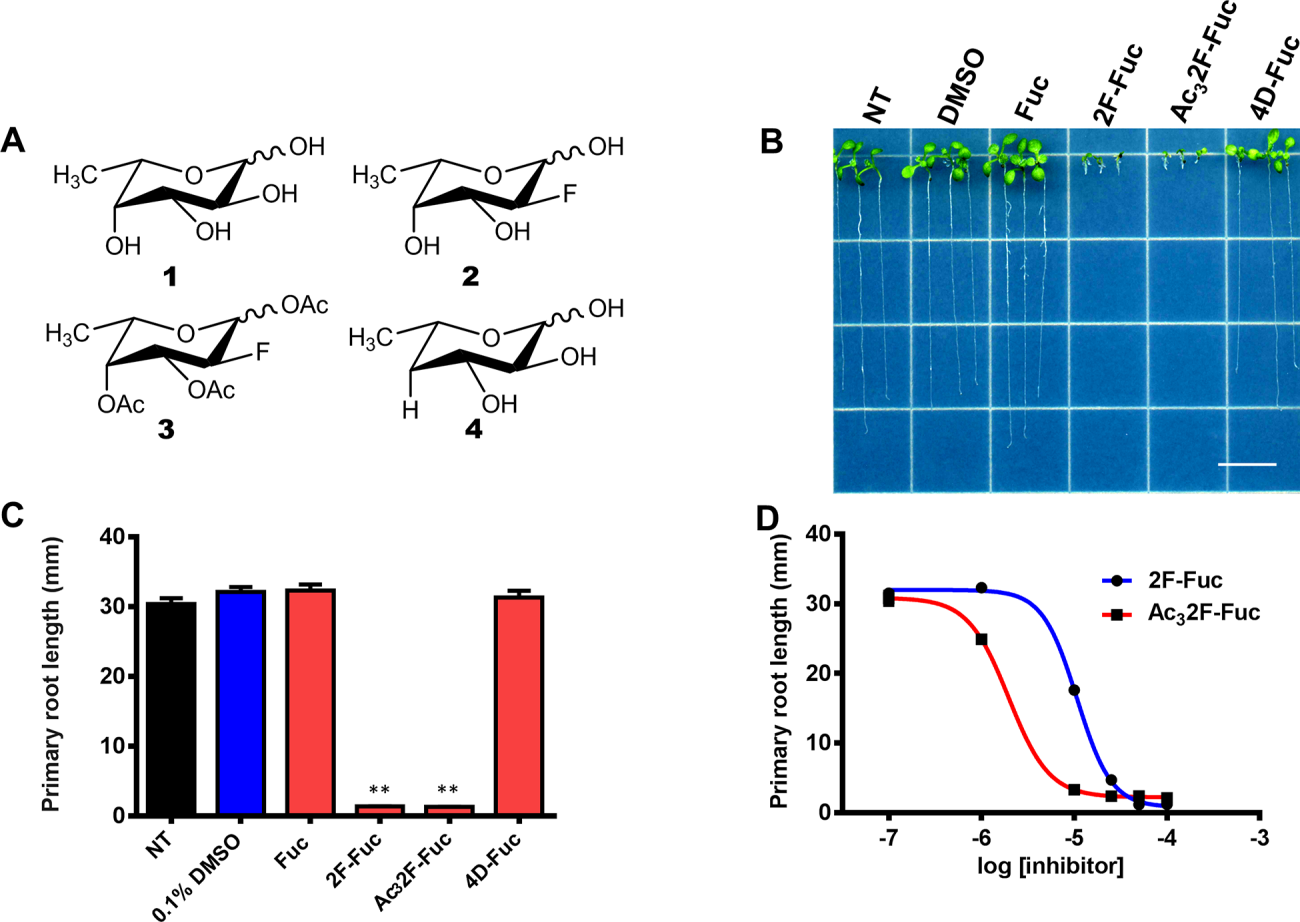
Effects of fluoro and deoxy L-Fuc analogs on Arabidopsis root growth. (A). The structures of L-fucose (1) as well as 2-fluoro-L-fucose (2), peracetylated 2-fluoro-L-fucose (3), and 4-deoxy-L-fucose (4) are shown. (B). Arabidopsis seedlings were grown for 7 days under long day conditions at 22°C on the media containing the following additives; from left to right: No treatment (NT), 0.1% DMSO (D), 100 μM L-Fuc (Fuc), 100 μM 2F-L-fucose (2F-Fuc), 100 μM Ac_3_2F-L-fucose (Ac_3_2F-Fuc), and 100 μM 4-Deoxy-L-fucose (4D-Fuc). The scale bar represents 1 cm. (C). Root length quantification measurements of 7-day-old seedlings grown on control media (black bar), media containing 0.1% DMSO (blue bar), or media containing 100 μM L-Fuc (Fuc), 2-fluoro-L-fucose (2F-Fuc), per-acetylated 2F-L-Fuc (Ac_3_2F-Fuc), and 4-deoxy-L-fucose (4D-Fuc) (red bars). Error bars represent SEM (n = 80–110). ** indicates P<0.0001, Student’s t-test. (D). Arabidopsis seedlings were grown for 7 days under constant light in the presence of increasing 2F-Fuc (blue line) or AC_3_2F-Fuc (red line) concentrations, and primary root lengths were quantified. The resulting data was fit to a single-exponential dose response curve. Error bars represent SEM (n = 80).

Characterized inhibitors of cell wall polysaccharide biosynthesis often produce clear phenotypic defects, including decreased cell expansion, loss of anisotropic cell elongation, and cell swelling [35–37, 39, 43, 44]. Therefore, we reasoned that if L-Fuc analogs inhibited cell wall biosynthesis, they would produce similar phenotypes. To investigate the phenotypic defects induced by flouro or deoxy L-Fuc analogs, three L-Fuc analogs were assayed (Fig 1A) for their ability to inhibit Arabidopsis root growth. L-Fuc analogs containing a fluorine substitution at the 2-position were chosen because fluorinated monosaccharides and their nucleotide sugar metabolites have been demonstrated to inhibit glycosylation events in mammalian systems [45, 46]. Additionally, 4-deoxy-L-Fuc (4D-Fuc) was assayed because the substitution of a hydride for a hydroxyl group could prevent glycosidic bond formation and thus serve as a polysaccharide chain terminator.

Arabidopsis seedlings were grown in the light for 7 days in the presence of these monosaccharide analogs to assess their effects. Seedlings grown on normal MS media or media supplemented with 100 μM L-Fuc were phenotypically indistinguishable. In contrast, the addition of 100 μM 2-fluoro-L-Fuc (2F-Fuc) or peracetylated-2-fluoro-L-Fuc (Ac_3_2F-Fuc) severely inhibited root growth by 95% compared to untreated plants (Fig 1B and 1C). Acetylated monosaccharides often exhibit increased membrane permeability, and the acetate groups are removed by non-specific esterases within the cytosol. Therefore, the potency of 2F-Fuc and Ac_3_2F-Fuc was compared by measuring root growth inhibition as a function of monosaccharide analog concentration. Fig 1D indicates that both 2F-Fuc and Ac_3_2F-Fuc inhibited Arabidopsis root growth in a dose-dependent manner, and IC_50_ values were calculated based on these dose response curves. 2F-Fuc exhibited a calculated IC_50_ of 10 μM, but Ac_3_2F-Fuc was more potent, with an IC_50_ of 2 μM. Overall, these results indicate the 2F-Fuc analogs are effective inhibitors of plant growth, and suggest that Ac_3_2F-Fuc may passively traverse the plasma membrane, producing a more potent inhibitor.

To investigate the short-term growth defects elicited by 2F-Fuc treatment, Arabidopsis seedlings were germinated on MS-agar medium and grown for 4 days at 22°C under long day conditions (16 hr light/ 8 hr dark). These seedlings were then transferred to new MS-agar media with or without the addition of 100 μM 2F-Fuc. Seedlings were grown vertically under the same conditions for an additional 3 days, and root lengths were quantified every 12 hours to investigate whether 2F-Fuc altered root elongation rates.

**Fig 2:**
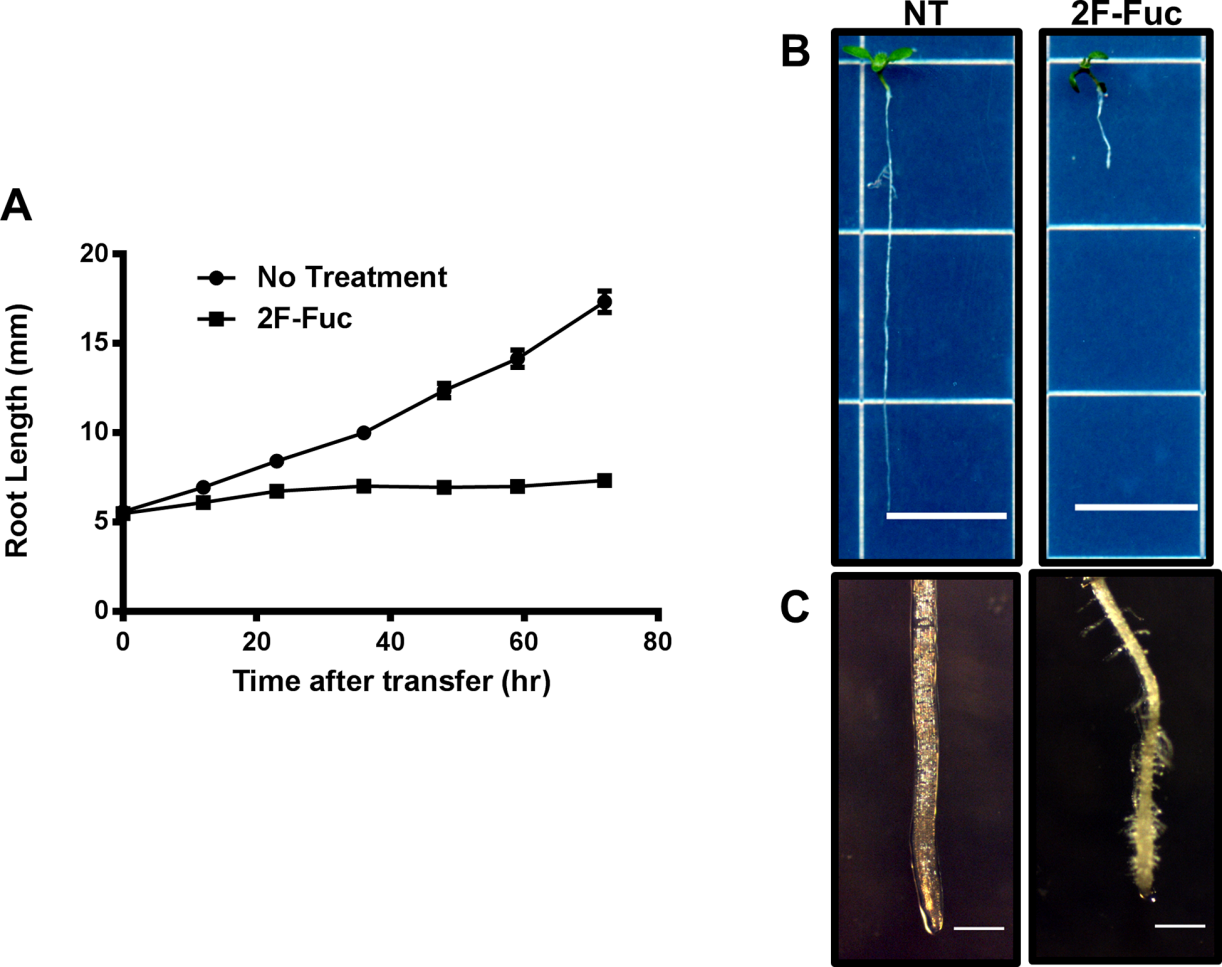
Short-term treatment effects on 2F-Fuc treated Arabidopsis seedlings. Arabidopsis Col-0 seedlings were grown under long day conditions at 22°C for 4 days in MS media before transferring to new media with or without 100 μM 2F-Fuc. Root lengths were measured every 12hrs for 3 days. (A). Average root length of seedlings transferred to MS media (NT) or media containing 100 μM 2F-Fuc (2F-Fuc) in 12hr increments. Error bars represent SEM (n = 48). (B). Representative 9-day-old untreated (NT) or 100 μM 2F-Fuc treated (2F-Fuc) seedlings. Scale bars represent 1cm. (C). Root tip images of 9-day-old seedlings in (B) under 35X magnification. Scale bar represents 0.5mm.

The results of this experiment are presented in Fig 2. Fig 2A illustrates that control seedlings transferred to media lacking 2F-Fuc continued to grow normally and exhibit a relatively linear root growth rate over the duration of the experiment. In contrast, seedlings transferred to media containing 100 μM 2F-Fuc exhibited severely reduced root elongation rates within 12 hours of transfer, and root elongation stopped completely within 24 hours of transfer. Overall seedling and root tip morphology was examined 5 days after transfer (Fig 2B and 2C). As observed in long-term treatments (Fig 1B and 1C), total root length was markedly reduced in 2F-Fuc treated seedlings compared to untreated controls. Additionally, the root tips of 2F-Fuc treated seedlings exhibited cell swelling, increased root tip diameter, and ectopic root hair production. Overall, these results suggest that 2F-Fuc mimics many of the effects elicited by known cell wall biosynthesis inhibitors and also demonstrates that 2F-Fuc effectively inhibits root growth under short-term treatment conditions.

### Genetic analysis indicates that 2F-Fuc analogs are metabolically incorporated through the fucose salvage pathway

**Fig 3:**
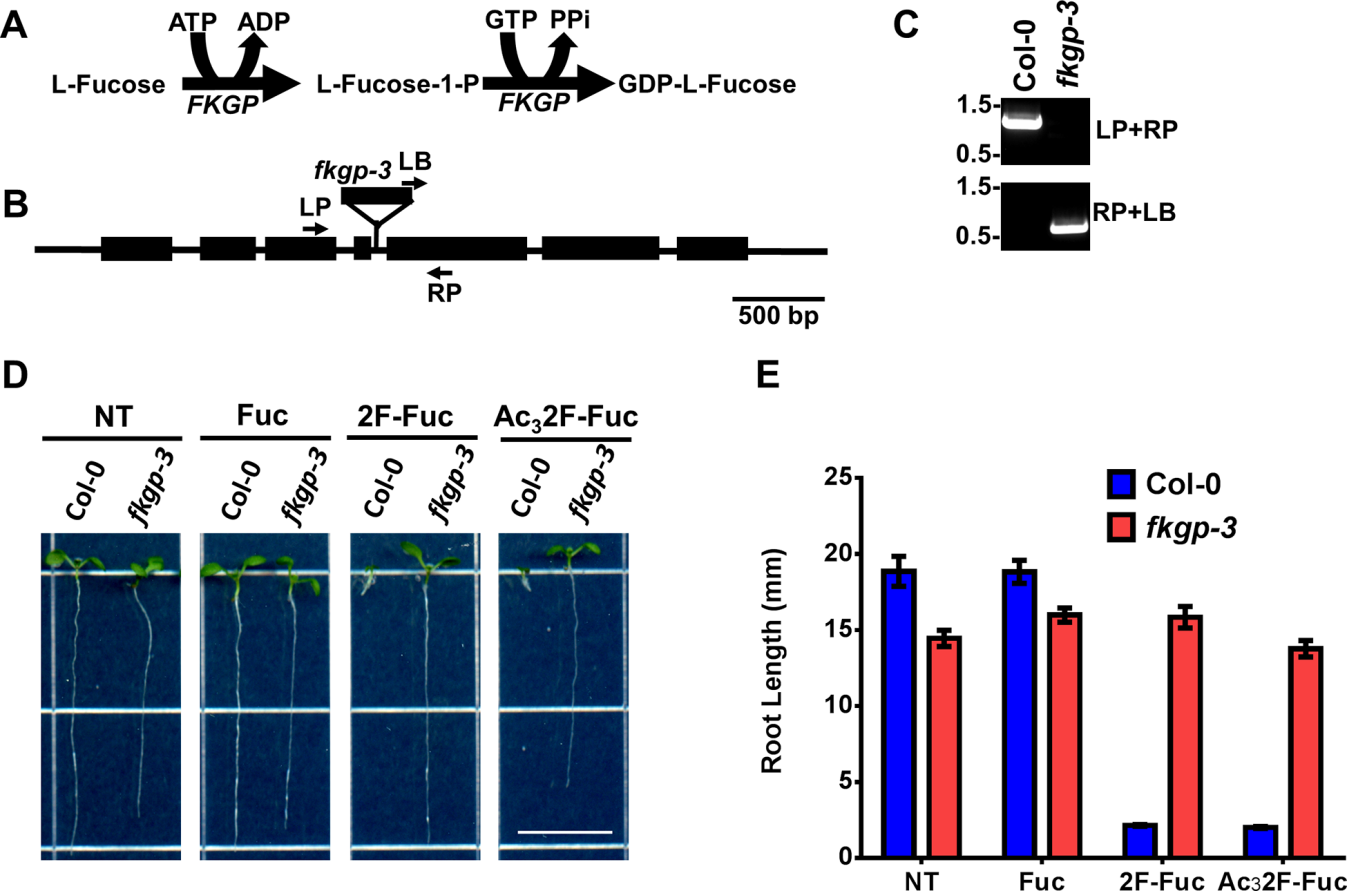
Identification of the fkgp-3 mutant and 2F-Fuc resistance assays. (A). The reactions catalyzed by the bifunctional FKGP enzyme are shown. FKGP phosphorylates L-Fuc at the anomeric hydroxyl group in an ATP-dependent manner to generate L-Fuc-1-phosphate (Fuc-1P). The GDP-L-Fuc pyrophosphorylase domain uses GTP and Fuc-1P to generate GDP-L-Fuc and pyrophosphate (PPi). (B). The genomic locus of the Arabidopsis FKGP gene (At1g01220) is shown, and the position of the *fkgp-3* T-DNA insertion is indicated. The position of the LP, RP, and LB primers used for PCR genotyping are also indicated. (C). PCR products from wild-type Col-0 and *fkgp-3* mutant genotyping PCR with LP+RP and RP+LB primer combinations were separated on a 1.0% agarose gel. The position of the 1.5 and 0.5 kb molecular weight markers are indicated. (D). Wild-type Col-0 and *fkgp-3* mutant seedlings were grown for 7 days under long day conditions at 22°C on MS media (NT) or MS media supplemented with 100 μM L-Fuc (Fuc), 2-fluoro-L-fucose (2F-Fuc), or per-acetylated 2-fluoro-L-fucose (Ac_3_2F-Fuc). Scale bar represents 1 cm. Root lengths from these seedlings were quantified (E) as described in Materials and Methods. Error bars represent SEM (n = 50).

Previous studies have indicated that monosaccharide analogs can impinge on cellular glycan metabolism in at least two distinct ways. These analogs can either inhibit enzymes responsible for monosaccharide biosynthesis [39] or can be converted to cognate sugar nucleotides via salvage pathway enzymes [46–48]. In Arabidopsis, the L-Fuc salvage pathway consists of a single bifunctional L-Fucose kinase/ GDP-L-Fucose pyrophosphorylase (FKGP) enzyme [21], which is responsible for salvaging all exogenously applied L-Fuc (Fig 3A). If fluorinated analogs are converted to GDP-2F-Fuc via the FKGP pathway and GDP-2F-Fuc is an active glycosyltransferase inhibitor, the Arabidopsis mutants lacking the FKGP gene should be resistant to 2F-Fuc analogs.

To test this hypothesis, the FKGP gene (At1g01220) was queried against the Arabidopsis T-DNA insertional mutant database (signal.salk.edu/cgi-bin/tdnaexpress), and potential T-DNA insertional mutants were obtained from the Arabidopsis Biological Resource Center (ABRC). Putative mutants were screened by PCR genotyping, and one *fkgp* mutant allele (SALK_037697) was confirmed to contain a T-DNA insertion in intron 4 of the FKGP gene (Fig 3B and 3C). Since two other *fkgp* T-DNA alleles were previously described [21], this allele was named *fkgp-3*. The *fkgp-3* mutant was grown on media containing 100 μM 2F-Fuc or 100 μM Ac_3_2F-Fuc in parallel with wild-type Col-0 controls, and Fig 3D and 3E illustrate the results of this experiment. When grown on media with no additives or media containing 100 μM L-Fuc the *fkgp-3* null mutant exhibited approximately 20% shorter primary roots than wild-type Col-0 controls. In contrast, 100 μM 2F-Fuc or 100 μM Ac_3_2F-Fuc severely inhibited primary root growth of Col-0 control plants, while the *fkgp-3* mutant seedlings were completely resistant to these treatments. These results suggest that FKGP activity is required for 2F-Fuc toxicity. Additionally, these results suggest that 2F-Fuc is metabolically converted to GDP-2F-Fuc, and that GDP-2F-Fuc is the active chemical species causing plant growth inhibition.

### Chemical analysis of monosaccharide abundance changes upon 2F-Fuc treatment

L-Fuc is a component of several cell wall polysaccharides and cellular glycans, including xyloglucan, pectic RG-I and RG-II, arabinogalactan proteins, and N-linked glycans. Potentially, 2F-Fuc could serve as a general inhibitor of cellular fucosylation events, or this molecule could target specific polysaccharides. To further investigate 2F-Fuc inhibition of L-Fuc incorporation into cell wall polysaccharides, the matrix polysaccharide composition of 7-day-old dark grown Arabidopsis Col-0 seedlings was determined by alditol acetate monosaccharide analysis [5, 6].

**Fig 4:**
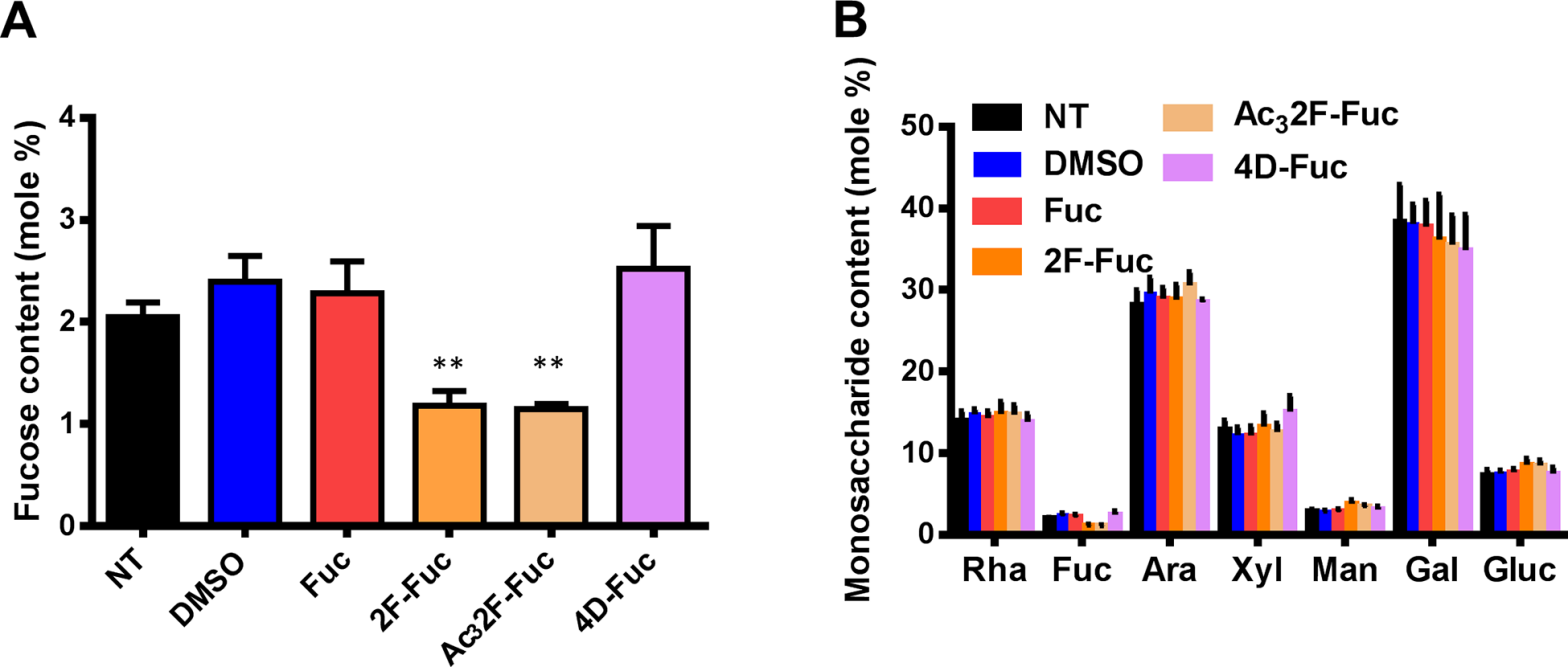
Monosaccharide analysis of matrix polysaccharides. Arabidopsis seedlings were grown on MS media without sucrose and containing the indicated additives in the dark at 22°C for 7 days. Alcohol insoluble residues (AIR) was prepared from these seedlings as described in Materials and Methods. Matrix polysaccharides were hydrolyzed to monosaccharides in 2M TFA, and these monosaccharides were derivatized to their corresponding alditol acetates for gas chromatography analysis. The (A) L-Fuc contents and (B) total cell wall monosaccharide contents of seedlings treated with 0.1% DMSO (blue bars), or 100 μM L-Fuc (Fuc; red bars), 2-fluoro-L-fucose (2F-Fuc; gold bars), per-acetylated 2-fluoro-L-fucose (Ac_3_2F-Fuc; tan bars), or 4-deoxy-L-fucose (4D-Fuc; purple bars) are shown. Error bars represent SEM (n = 5). ** indicates P <0.005, Student’s t-test.

Arabidopsis seedlings were grown on media with or without L-Fuc or fucose analogs. The seedlings were harvested, and alcohol insoluble residue (AIR) was prepared as described in Materials and Methods. The resulting AIR fraction was hydrolyzed with aqueous TFA to release monosaccharides from matrix polysaccharides, and the resulting monosaccharides were derivatized to their corresponding alditol acetates for gas chromatography analysis. Untreated or solvent control plants exhibited cell wall monosaccharide compositions similar to those previously reported for Arabidopsis. In contrast, Arabidopsis seedlings treated with 100 μM 2F-Fuc or 100 μM Ac_3_2F-Fuc exhibited a 50% reduction in matrix polysaccharide L-Fuc content (Fig 4A). The remainder of the cell wall monosaccharide contents in these samples were unchanged compared to untreated or solvent control plants (Fig 4B). Additionally, plants treated with 4D-Fuc exhibited no change in matrix polysaccharide L-Fuc content, suggesting that the observed effect is specific for 2F-Fuc. These results indicate that 2F-Fuc treatment specifically reduces cell wall L-Fuc content without substantially altering the abundance of other cell wall monosaccharides, suggesting that GDP-2F-Fuc specifically inhibits cellular fucosyltransferases.

### Genetic analysis of fucosyltransferase knockouts suggest that 2F-Fuc is not incorporated into cell wall polysaccharides

Two possible scenarios could account for the phenotypic defects resulting from 2F-Fuc treatment. First, the monosaccharide could be incorporated into cell wall polysaccharides through the action of cognate glycosyltransferases, where the fluorinated analog could alter the structure of these polysaccharides or molecular interactions between polysaccharides. Alternatively, 2F-Fuc or its nucleotide sugar metabolite GDP-2F-Fuc could directly inhibit glycosyltransferases involved in cell wall polysaccharide biosynthesis and prevent L-Fuc incorporation into cell wall polysaccharides. Fucosyltransferases for xyloglucan [18], arabinogalactan proteins [14, 15], and N-linked glycans [16] have been identified and characterized in Arabidopsis. If incorporation of 2F-Fuc into a particular glycan altered molecular interactions with other cell wall polysaccharides, then mutating the cognate fucosyltransferase would generate resistance to 2F-Fuc. Alternatively, if inhibition of a specific fucosylation event is responsible for the observed 2F-Fuc growth defects, then mutating the responsible fucosyltransferase should phenocopy the growth defects associated with 2F-Fuc treatment.

**Fig 5:**
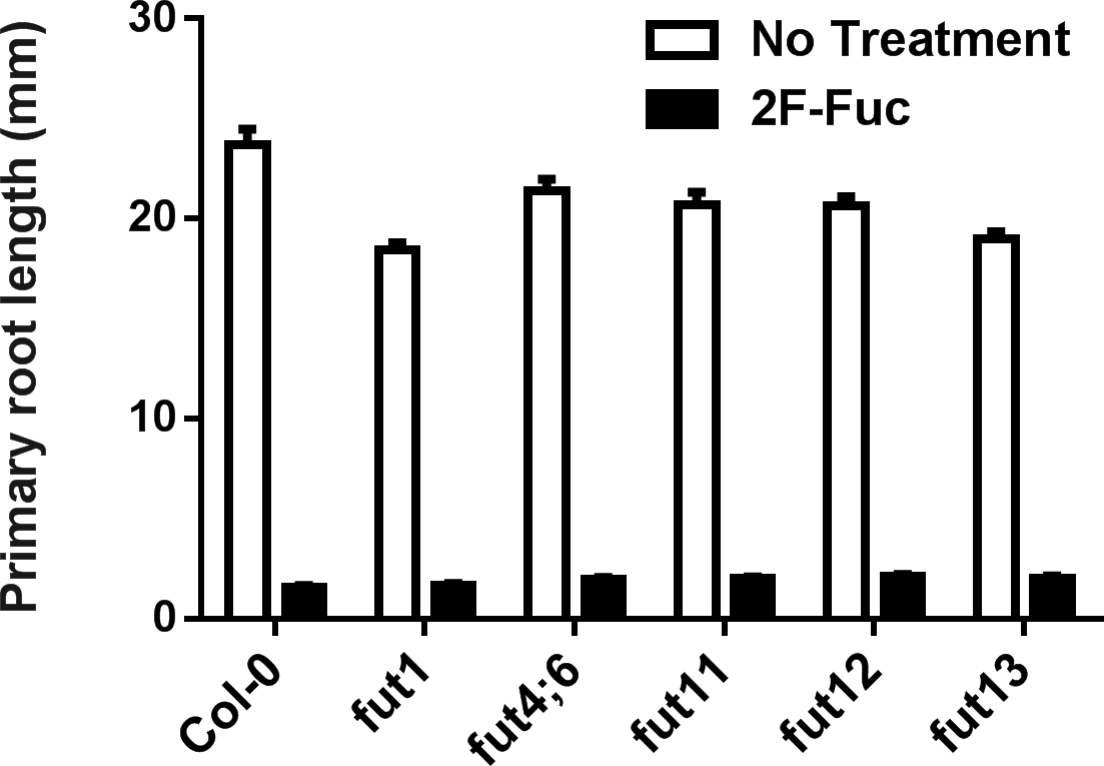
Analysis of fucosyltransferase mutant 2F-Fuc sensitivity. Arabidopsis Col-0 seedlings were grown on MS media with (black bars) or without (white bars) the addition of 100 μM 2F-Fuc in parallel with *fut1* (SALK_139678), *fut4;6* double knockout, *fut11* (SALK_134085), *fut12* (SALK_063355), and *fut13* (SALK_067444) mutants. After 7 days of growth at 22°C under long day conditions, primary root lengths were quantified as described in Materials and Methods. Error bars represent SEM (n = 30).

Previously characterized Arabidopsis T-DNA insertional mutants in the xyloglucan fucosyltransferase FUT1 [18], the arabinogalactan protein fucosyltranferase FUT4 and FUT6 [14, 15], and the N-linked glycan fucosyltransferases FUT11, FUT12, and FUT13 [16, 40] were propagated on MS media with or without 100 μM 2F-Fuc. Primary root lengths of each line were measured after 7 days of growth (Fig 5). All fucosyltransferase knockout lines exhibited similar root lengths to wild-type Col-0 plants on normal media, and similar root growth inhibition on media containing 100 μM 2F-Fuc. These results suggest that the phenotypic defects observed after 2F-Fuc treatment are not the result of incorporation of this monosaccharide analog into xyloglucan, arabinogalactan proteins, or N-linked glycans. Additionally, the phenotypes of these mutants on control media suggest that inhibition of specific fucosylation events in xyloglucan, arabinogalactan proteins, or N-linked glycans cannot explain the 2F-Fuc phenotypic defects.

### 2F-Fuc phenotypic defects can be partially complemented by exogeneously applied boric acid

Genetic lesions in the Arabidopsis MUR1 gene cause severe growth defects in aerial tissues, including reduced cell expansion and altered leaf morphology [22], that were linked to altered fucosylation in RG-II [24, 26]. RG-II also uniquely contains Kdo, and recently, a small molecule inhibitor of Arabidopsis CMP-Kdo synthase was developed. 2β-deoxy-Kdo inhibited Arabidopsis root elongation, and plants treated with this compound exhibited marked cell swelling at the root tip [39]. These effects, and the growth defects in the aerial tissues of the *mur1* mutant could be partially rescued by the exogenous application of boric acid [26, 39].

**Fig 6:**
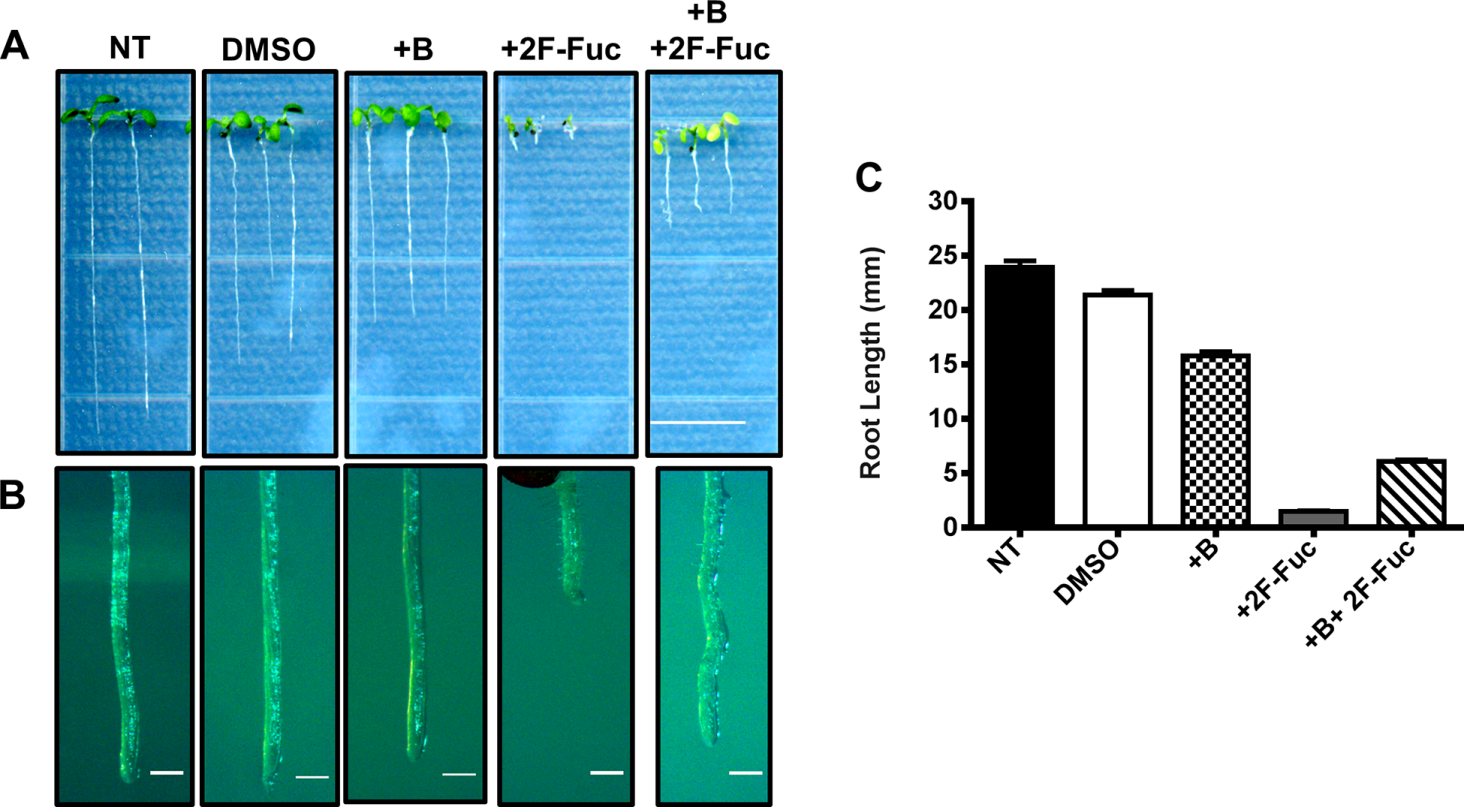
Chemical complementation of 2F-Fuc growth defects with boric acid. (A). Arabidopsis Col-0 seedlings were grown on MS media containing 0.1% DMSO (DMSO), 1.5 mM boric acid (+B), 100 μM 2-fluoro-L-fucose (2F-Fuc), or a combination of 1.5 mM boric acid and 100 μM 2-fluoro-L-fucose (+B + 2F-Fuc) for 7 days in constant light at 22°C. Col-0 seedlings grown under the same conditions with MS media containing no additives served as a negative control (NT). Scale bar represents 1 cm. (B). Higher magnification images of primary root tips for each treatment are shown. Scale bar represents 250 μm. (C). Primary root lengths for each treatment were quantified as described in Materials and Methods. Error bars represent SEM (n = 30).

To investigate whether 2F-Fuc potentially affects RG-II biosynthesis, we determined whether the growth defects resulting from 2F-Fuc treatment could be rescued by boric acid treatment. Arabidopsis seedlings were grown on MS media containing 100 μM 2F-Fuc, 1.5 mM boric acid, or the combination of these additives. Plants were grown for seven days and their root lengths were quantified. Fig 6 illustrates Arabidopsis roots on 1.5 mM Boric acid containing media were 30% shorter than wild-type untreated plants, but plants treated with 100 μM 2F-Fuc were approximately 95% shorter than untreated controls. 2F-Fuc treatment also resulted in altered root morphology (Fig 6B). Seedlings treated with both 100 μM 2F-Fuc and 1.5 mM boric acid partially rescued both the root elongation defect and root morphology phenotypes (Fig 6A and 6C), indicating that 2F-Fuc treatment elicits similar effects to the previously described inhibitor of CMP-Kdo synthase. These results suggest that 2F-Fuc inhibits the synthesis of RG-II, most likely side chains A and B which contain L-Fuc.

## Discussion

L-Fuc is an important component of plant cell wall polysaccharides and other cellular glycans. The *mur1* Arabidopsis mutant highlights the importance of this monosaccharide in plant growth and development. These mutants exhibit reduced cell expansion in aerial tissues, obvious dwarfism, and reduced cell wall matrix polysaccharide L-Fuc content [19, 22]. Additionally, pectic RG-II structure and function is altered in the *mur1* mutant. Fucose residues normally occurring in the RG-II A and B side chains are replaced with L-galactose, and these substitutions inhibit RG-II dimer formation [24]. Exogeneously applied boric acid can partially or completely relieve the phenotypic defects observed in *mur1* aerial tissues [26, 39]. While considerable efforts have led to the detailed structural characterization of RG-II from a variety of plant species, it is still unclear how this polysaccharide is synthesized and what precise role RG-II plays in the modulation of cell wall structure during plant growth and development. Therefore, a library of small molecule probes that can be utilized to perturb RG-II structure would be extremely useful.

In this work, we demonstrate that 2F-Fuc and its per-acetylated analog Ac_3_2F-Fuc cause extreme dwarfism, altered root morphology, and reduced cell wall L-Fuc content (Figs 1, 2 and 4) when applied to Arabidopsis seedlings. Furthermore, many of these phenotypic defects could be partially rescued by the exogeneous application of boric acid (Fig 6). While L-Fuc is a component of several cell wall polysaccharides and cellular glycans [10, 14–16, 18, 40], loss-of-function mutations in the glycosyltransferases responsible for these fucosylation events did not mimic phenotypes associated with 2F-Fuc treatment (Fig 5). Compared to wild-type Col-0 plants, these loss-of-function mutants were also equally sensitive to 2F-Fuc. These results suggest that 2F-Fuc cannot be incorporated into fucosylated glycans to disrupt their structure or interaction with other cellular components because loss of the cognate fucosyltranferase should disrupt this process and result in 2F-Fuc resistance. Root elongation and morphology of these fucosyltransferase mutants on media lacking 2F-Fuc also suggests that loss of xyloglucan, arabinogalactan protein, or N-linked glycan fucosylation cannot explain the growth defects observed upon 2F-Fuc treatment.

In Arabidopsis, the bifunctional enzyme FKGP converts free L-Fuc to GDP-L-Fuc in two steps [21]. The demonstration that the *fkgp-3* loss-of-function mutant is resistant to 2F-Fuc toxicity (Fig 3) suggests that FKGP activity is required to produce these phenotypic effects. The simplest hypothesis to explain these observations is that FKGP converts 2F-Fuc to GDP-2F-Fuc through the consecutive action of its L-Fuc kinase and GDP-L-Fuc pyrophosphorylase activities. All known glycosyltransferases use sugar nucleotides, and not isolated monosaccharides, as substrates. Therefore, we suggest a model in which 2F-Fuc is metabolically converted to GDP-2F-Fuc through the FKGP enzyme, and GDP-2F-Fuc serves as a sugar nucleotide-specific glycosyltransferase inhibitor. This conversion to a sugar nucleotide form has been observed for a variety of monosaccharide analogs. For example, alkynyl or azido monosaccharides have been widely utilized as chemical probes to label cellular glycans [40, 47–50]. These monosaccharides are converted to sugar nucleotides through the activity of cognate salvage enzymes [47, 48]. Subsequently, the alkynyl or azido monosaccharide analog is transferred to a specific glycan through the action of cognate glycosyltransferases, and the alkynyl or azido group is bio-orthogonally labeled by the copper catalyzed click reaction [40, 49, 50].

Additionally, fluorinated monosaccharides have been demonstrated to serve as useful glycosyltransferase inhibitors [45, 46, 51, 52]. For example, 4-fluoro-D-glucosamine (4F-GlcNAc) was demonstrated to inhibit the biosynthesis of N-linked glycans in animal cells [45]. This monosaccharide analog was not incorporated into newly synthesized N-linked glycans, but prevented the incorporation of its parent monosaccharide, suggesting that 4F-GlcNAc inhibits the glycosyltransferase activity of the cognate N-glycan GlcNAc glycosyltransferase. Additionally, 2-fluoro derivatives of L-Fuc and sialic acid were recently demonstrated to inhibit mammalian glycosyltransferases involved in transferring L-Fuc and sialic acid to N-linked glycans [46], suggesting that fluorinated monosaccharides could be generally useful probes for the investigation of cell wall polysaccharide biosynthesis.

Many glycosyltransferases form a transition state in which the enzyme binds a sugar nucleotide and acceptor substrate. In this transition state, the nucleotide moves away from the donor monosaccharide, creating a partial positive charge at the anomeric carbon, which is utilized as an attack point by the donor hydroxyl [52]. Due to its extreme electrophilic character, a fluorine substitution at the 2-position would create a partial positive charge adjacent to the anomeric carbon, resulting in two repelling partial positive charges and destabilization of the glycosyltransferase transition state complex. This phenomenon potentially represents the chemical basis for the inhibition of glycosyltransferase by fluorinated sugar nucleotides. Therefore, it is likely that 2-fluoro monosaccharide analogs could represent rationally designed glycosyltranferase inhibitors for the manipulation of plant cell wall polysaccharide biosynthesis. It will be interesting to investigate whether other 2-fluoro monosaccharide analogs exhibit similar effects on plant cell wall biosynthesis in the future.

Based on the observation that exogeneously applied boric acid partially complements the phenotypic defects induced by 2F-Fuc, we hypothesize that 2F-Fuc serves as a global metabolic inhibitor of fucosylation events, but the primary growth inhibitory effect results from disruption of RG-II biosynthesis. RG-II is a complex polysaccharide that contains 12 unique monosaccharides in 20 different linkages [23]. However, the structure of RG-II is remarkably conserved amongst plant species, suggesting that the function of this polysaccharide is also highly conserved. While a great deal of work has focused on elucidating the structural details of RG-II, the enzymes involved in RG-II biosynthesis remain largely unknown [53]. RG-II uniquely contains the acidic monosaccharide Kdo, and this monosaccharide is also observed in bacterial lipid A [54, 55]. In both organisms, Kdo is converted to cytidine monophosphate-Kdo (CMP-Kdo) in a reaction catalyzed by CMP-Kdo synthase or KdsB [55]. The Arabidopsis genome contains a single KdsB isoform that was demonstrated to produce CMP-Kdo *in vitro*. However, homozygous loss-of-function mutations in this gene were never recovered due to gametophytic lethality [29]. Similar observations indicate that putative RG-II Kdo, 2-keto-3-deoxy-D-*lyxo*-heptulosaric acid (Dha), and xylose glycosyltransferases exhibit similar gametophytic lethality due to impaired pollen tube growth [30–33]. These observations suggest that disrupting RG-II structure severely compromises plant reproduction, precluding the analysis of homozygous loss-of-function mutants at the seedling or adult growth stages. Therefore, well characterized inhibitors of RG-II biosynthesis, such as 2β-deoxy-Kdo [39] will be essential tools that can be utilized to dissect RG-II function. Based on the observations presented here, 2F-Fuc may represent an additional chemical probe for RG-II biosynthesis inhibition as well as other cellular fucosylation events.

Interestingly, RG-II contains two central fucose residues in side chains A and B [12, 13]. Although the Arabidopsis genome contains 10 FUT genes similar to the xyloglucan fucosyltransferase FUT1 [17], none of these genes have been implicated in RG-II fucosylation. Potentially, one or several of these fucosyltransferases also result in gametophytic lethality due to impaired RG-II biosynthesis, and this will be an interesting research avenue to pursue in the future. Based on the results presented here, partial loss-of-function mutations would be expected to phenocopy 2F-Fuc treatment, highlighting the importance of small molecule inhibitors in the discovery of new genes involved in cell wall biosynthesis. In summary, we provide evidence that 2F-Fuc is converted to GDP-2F-Fuc via plant cellular metabolism, and that this molecule serves as an inhibitor of Arabidopsis fucosyltransferases. In the future, it will be useful to investigate whether other 2-fluorinated monosaccharides inhibit a unique set of glycosyltransferases and whether these monosaccharide analogs impinge upon cell wall polysaccharide biosynthesis.
